# Knowledge, attitude, and practice of patients with type 2 diabetes mellitus with regard to their disease: a cross-sectional study among Palestinians of the West Bank

**DOI:** 10.1186/s12889-021-10524-2

**Published:** 2021-03-09

**Authors:** Ramzi Shawahna, Saed Samaro, Zaid Ahmad

**Affiliations:** 1 Department of Physiology, Pharmacology and Toxicology, Faculty of Medicine & Health Sciences, New Campus, Building: 19, Office: 1340, An-Najah National University, P.O. Box 7, Nablus, Palestine; 2 An-Najah BioSciences Unit, Centre for Poisons Control, Chemical and Biological Analyses, An-Najah National University, Nablus, Palestine; 3 Department of Medicine, Faculty of Medicine and Health Sciences, An-Najah National University, Nablus, Palestine

**Keywords:** Associated factors, Attitude, Diabetes mellitus, Knowledge, Palestine, Practices

## Abstract

**Background:**

In Palestine, type 2 diabetes mellitus (T2DM) is a rapidly growing health concern. This study evaluated knowledge, attitude, and practice of patients with T2DM with regard to their disease. The study also investigated association and correlation between knowledge, attitude, and practice with sociodemographic and clinical characteristics of the patients. Predictors of higher knowledge, positive attitude, and good practice were also identified.

**Methods:**

This cross-sectional study was conducted in primary healthcare facilities frequently visited by patients with T2DM across the West Bank of Palestine in the period of October 2018 to January 2019. An interviewer administered questionnaire was used to determine knowledge, attitude and practice of patients with T2DM with regard to their disease.

**Results:**

Out of 300 patients invited, 220 (73.3%) patients responded. In this study, the median age was 57.0 years (51.0, 65.0), the median time elapsed since diagnosis with T2DM was 7.0 years (4.0, 14.0), the median fasting blood glucose was 150.0 mg/dL (128.8, 180.0), the median postprandial glucose was 230.0 mg/dL (200.0, 270.0), the median HbA_1c_ was 7.8% (7.0, 8.53), and the median BMI was 28.8 kg/m^2^ (25.5, 33.1). The median knowledge score was 6.0/13.0 (4.5/13.0, 7.5/13.0), the median attitude score was 3.0/4.0 (2.0/4.0, 4.0/4.0), and the median practice score was 3.0 (1.0/5.0, 4.0/5.0). Having university education was strongly associated with having higher knowledge scores (*p*-value = 0.001). Additionally, having attended an educational program on diabetes was moderately associated with higher practice scores (*p*-value = 0.026).

**Conclusions:**

Findings of this study highlighted the need for appropriately designed interventions to increase knowledge about T2DM among patients with low educational level. Well-designed educational programs might promote healthy practice among patients with T2DM. Future studies are still needed to assess if such interventions could be effective in improving health outcomes and quality of life of patients with T2DM in Palestine.

## Background

Type 2 diabetes mellitus (T2DM) is a major metabolic disease that is characterized by sustained high levels of blood glucose which result from defective secretion of insulin, ineffective insulin action, or both [1, 2]. Globally, T2DM is considered a major public health epidemic with considerable number of premature deaths, disabilities, high morbidity, and mortality rates [3]. According to recent estimates, about 462 million people were affected by T2DM in 2017 which corresponded to approximately 6.3% of the world’s population [4]. Today, T2DM is the 9th leading cause of mortality that claims 1 million human lives on an annual basis. In Palestine, T2DM is a rapidly growing health concern. The prevalence of T2DM in the general Palestinian population was estimated at 15.3%. The prevalence rate was forecasted to increase to 20.8% for 2020 and 23.4% for 2030 [5].

Complications associated with T2DM include hypoglycemia, hyperosmolar hyperglycemic state, diabetic ketoacidosis, retinopathy, nephropathy, neuropathy, and cardiovascular events [6, 7]. Today, T2DM is a major cause of lower limb amputations [8, 9]. Interestingly, complications associated with T2DM might be mitigated by adherence to proper management approaches which aim to achieve optimal glycemic control and/or decrease macro- and microvascular complications of T2DM [10–12]. Management of T2DM involves both lifestyle modifications and pharmacological therapy [13]. Achieving optimized patient health outcomes requires high levels of knowledge of the disease, access to insulin and other oral hypoglycemic agents, and equipment to monitor blood glucose [14].

Recent studies that were conducted to assess knowledge, attitude, and practice of patients with T2DM highlighted the necessity of higher awareness among patients with regard to preventing, diagnosis, mitigating risk factors, and minimizing complications of the disease [15–18]. It has been argued that educating patients on their disease was an effective strategy to reduce complications of T2DM and achieve improved control over blood glucose [19, 20]. Probably, higher knowledge could improve attitude and practice of the patients with regard to their disease. Previous studies have shown that patients who were informed about their T2DM and the complications associated with the disease reported improved adherence to treatment compared to patients who had poor knowledge of T2DM and the complications associated with the disease. It has been argued that patients with T2DM who are knowledgeable of their disease would achieve better control of their blood glucose [21]. This might improve the health and quality of life of the patients. Additionally, unfavorable attitudes were associated with poor control over blood glucose and incidence of complications [22]. Previous studies have shown differences in knowledge, attitude, and practice among patients with T2DM in primary and tertiary healthcare settings in Saudi Arabia, Sri Lanka, Ethiopia, and India [3, 15–18].

Although having good knowledge, attitude, and practice regarding T2DM would be helpful in improving patient related health outcomes, currently, little is known on the level of knowledge, attitude, and practice among Palestinians with T2DM with regard to their disease. Therefore, this study was conducted to evaluate knowledge, attitude, and practice among Palestinians with T2DM in the West Bank. The study also investigated association and correlation between knowledge, attitude, and practice with sociodemographic and clinical characteristics of the patients. Predictors of higher knowledge, positive attitude, and good practice were also identified. Findings of this study could inform future decisions and interventions to improve control over blood glucose, reduce the incidence of complications, improve care, and quality of life of Palestinians with T2DM.

## Methods

### Study settings and design

This study was conducted in different primary healthcare facilities that were frequently visited by patients with T2DM across the West Bank of Palestine. Patients were approached and invited to take part in the study by the field researchers. The sample was collected in the period of October 2018 to January 2019 from 9 of the 11 (81.8%) primary healthcare facilities in the different governorates of the West Bank of Palestine. The field researchers could not visit and recruit patients from the governorates of Jerusalem and Hebron due to logistic reasons. After obtaining their consent, patients with T2DM were interviewed to determine their knowledge, attitude, and practice toward T2DM. This study was conducted in a cross-sectional design using a validated and reliable study tool. The study tool was tested for reliability in a pilot before conducting the larger study.

### Population, inclusion, and exclusion criteria

The study population was patients with T2DM who visited primary healthcare facilities in the West Bank of Palestine. Patients of both genders who were at least 18 years old and diagnosed with T2DM at least 6 months ago were invited to take part in this study. Patients were included if they provided verbal consent and were willing to respond to questions in a questionnaire.

### Sample size and sampling method

According to the International Diabetes Federation, there were 174,300 patients with T2DM in Palestine [23]. The sample size required for this study was calculated using an online sample size calculator (http://www.raosoft.com/samplesize.html). The sample size was estimated for a population of more than 20,000 with a 95% confidence interval and accepting a 5% margin of error. The sample size required for this study was 243 patients with T2DM. A convenience sampling method was followed to recruit participants in this study. Patients with T2DM were approached and recruited by field researchers from different primary healthcare facilities in the West Bank of Palestine.

### Study tool

The study was conducted using a pre-validated and reliable questionnaire to collect data relevant to knowledge, attitude, and practice of patients with T2DM with regard to their disease. The questions included in the questionnaire were informed by relevant literature [24–26]. The questionnaire collected sociodemographic characteristics of the patients like age, gender, body weight, height, marital status, employment, place of residence, educational level, and monthly income. The body weight and height were used to calculate the body mass index (BMI) of the patient [27]. Patient health records were used to obtain the time elapsed since they received their diagnosis with T2DM, if they had a health insurance, if they have had attended an educational program regarding diabetes, their usual fasting plasma glucose level, their usual postprandial plasma glucose level, and their usual glycated hemoglobin (HbA_1c_) level. Regarding knowledge, the patients were asked to answer the items regardless of their actual practice. The patients were asked to specify the tests used to diagnose DM, what can be done to keep DM under control, how long patients should continue adhering to diet control/ treatment, and what organs would DM affect. Regarding attitude, the patients were asked to answer the items regardless of their practice. Attitude items included whether occasional eating of sweets was alright, forgetting to take insulin or antidiabetic medications on some says was alright, whether not practicing the recommended exercise was alright, and whether the patients should go for regular checkups even if their blood glucose was under control. Regarding practice, the patients were asked to answer items relevant to their actual practice. Practice items included whether the patients: took insulin and/or other antidiabetic medications as their caring physicians recommended, followed diet schedule as recommended by their caring healthcare providers, adhered to practicing regular exercise as recommended by their caring healthcare providers, whether their blood glucose was under control, and whether they regularly go for follow ups with their caring healthcare professionals.

The data were collected into an interviewer administered questionnaire in face-to-face interviews. The interviewers were final year Doctor of Medicine (MD) candidates who were trained to administer interviews.

### Face validity, pilot testing, reliability and internal consistency

The study tool was reviewed by 5 researchers who had experience in conducting research on T2DM. The researchers who were physicians (*n* = 3) and pharmacists (*n* = 2) rated each item for relevance using a Likert-scale of 1–5 (1 = not relevant at all, 5 = highly relevant). Items that were rated as not relevant at all or not relevant by all researchers were excluded. Items that were rated as relevant or highly relevant by all researchers were retained. Equivocal items were resolved by discussion and consensus.

The study tool was pilot tested with 25 patients to assess if the questions were readable and understandable. To test the stability of the scores over a short period of time, the test-retest method was used. The 25 patients were asked to answer the items twice. A short time interval (30 min to 1 h) was let between both rounds. Scores obtained in the two rounds were correlated using Pearson’s correlation. Excellent stability of scores was indicated by a Pearson’s correlation coefficient of 95% (95% CI = 91.2 to 98.7%) with a *p*-value of < 0.001. As used in previous studies, acceptable coefficients were set a priori as > 80% [28–32]. Cronbach’s alpha statistics were used to assess the internal consistency of the items used in the study tool. Internal consistency of the items used in the test was good as indicated by a Cronbach’s alpha of 74.2%. Acceptable coefficients were set a priori between 70 and 95%.

### Data analysis

For knowledge, attitude, and practice items, the patients were given 1 point for each correct/positive answer and 0 for each incorrect/negative answer. Knowledge scores could range from 0 to 13, attitude scores could range from 0 to 4, and practice scores could range from 0 to 5.

The data obtained in this study were coded and entered into IBM SPSS for Windows v.21.0 0 (IBM Inc., Armonk, New York) for analysis. To determine whether the data were normally distributed or not, Shapiro–Wilk test was used. As determined by the test, the data were not normally distributed. Therefore, the 1st quartile (Q1), 2nd quartile (Q2 = median), and 3rd quartile (Q3) were used to express the data. The variables: age, time elapsed since diagnosis, BMI, fasting plasma glucose, postprandial plasma glucose, and HbA_1c_ were analyzed as continuous and categorical variables. Categorical data were compared using the Mann-Whitney *U* test or Kruskal-Wallis test. Spearman’s rank correlation was used to assess correlations between variables. To control confounding factors, we conducted a multiple linear regression. All variables that were used in the Mann-Whitney *U* test and Kruskal-Wallis test were retained in the model. The variables: age, time elapsed since diagnosis, BMI, fasting plasma glucose, postprandial plasma glucose, and HbA_1c_ were included as continuous variables and the coefficients reported were per unit change. However, the variables: gender, marital status, employment, place of residence, educational level, monthly income, having a health insurance, and having attended an educational program were included as categorical variables. Female, single, unemployed, urban, some school, lower income, not having a health insurance, and not having attended an educational program were the reference categories for gender, marital status, employment, place of residence, educational level, monthly income, having a health insurance, and having attended an educational program, respectively. Variance inflation factor (VIF) and tolerance values were used to evaluate collinearity diagnostics. VIF values of < 1.76 and tolerance values of > 1.01 indicated absence of multicollinearity issues [33, 34]. In this study, a *p*-value of < 0.05 was considered statistically significant. The adjusted R^2^ with a *p*-value of < 0.05 was used to evaluate the goodness of fit of the regression model.

### Ethics approval and consent to participate

Ethics approval was obtained from the Institutional Board Review (IRB) of An-Najah National University. Field researchers explained the study design and objectives to the potential participants and obtained their written informed consent before they took part in the present study. The IRB of An-Najah National University approved a verbal consent for this study. Approval was obtained from the Ministry of Health to access the healthcare centers.

## Results

### Characteristics of the patients

Of the 300 patients with T2DM who were initially invited, 220 (73.3%) patients completed the questionnaire. Of the study patients, 138 (62.7%) were 55 years of age and older, 118 (53.6%) were female, 156 (70.9%) were married, 139 (63.2%) were unemployed, 149 (67.7%) resided in urban areas, 55 (25.0%) has university degrees, 135 (61.4%) had moderate or high income, 116 (52.7%) were diagnosed with T2DM 7 or more years ago, 45 (20.5%) did not have any health insurance, 198 (90.0%) did not attend any educational program regarding DM, 154 (70.0%) had their usual fasting plasma glucose level of more than 140 mg/dL, 177 (80.5%) had their usual postprandial plasma glucose level of more than 200 mg/dL, 167 (75.9%) had their HbA_1c_ level more than 7%, and 175 (79.5%) had a BMI of more than 25 kg/m^2^. Male and female patients significantly differed in relation to their marital status (*p*-value = 0.001), employment status (*p*-value < 0.001), and income level (*p*-value = 0.001). The median age of the patients was 57.0 (51.0, 65.0) years. The median time elapsed since diagnosis with T2DM was 7.0 (4.0, 14.0) years. The median fasting blood glucose was 150.0 (128.8, 180.0) mg/dL. The median postprandial glucose was 230.0 (200.0, 270.0) mg/dL. The median HbA_1c_ was 7.8% (7.0, 8.53). The median BMI was 28.8 (25.5, 33.1) kg/m^2^. There were no statistically significant differences between the continuous sociodemographic and clinical characteristics of the male and female patients (*p*-value > 0.05). Details of the sociodemographic and clinical variables of the patients overall and stratified by gender are shown in Table 1.

**Table 1:** Detailed sociodemographic and clinical characteristics of the patients overall and stratified by gender (*n = 220*)

Variable	Total		Male		Female		***p***-value
	N	%	n	%	n	%	
**Age (years)**							
< 55	82	37.3	36	35.3	46	39.0	0.580
≥ 55	138	62.7	66	64.7	72	61.0	
**Median (Q1, Q3)**			58.5 (50.0, 66.0)		56.0 (52.0, 64.0)		0.598
**Marital status**							
Single	32	14.5	8	7.8	24	20.3	0.001
Married	156	70.9	85	83.3	71	60.2	
Divorced/widowed	32	14.5	9	8.8	23	19.5	
**Employment**							
Unemployed	139	63.2	38	37.3	101	85.6	< 0.001
Employed	81	36.8	64	62.7	17	14.4	
**Place of residence**							
Urban	149	67.7	72	70.6	77	65.3	0.470
Country side	71	32.3	30	29.4	41	34.7	
**Educational level**							
Some school	165	75.0	75	73.5	90	76.3	0.755
University	55	25.0	27	26.5	28	23.7	
**Monthly household income (Jordanian Dinar)**							
< 600	85	38.6	26	25.5	59	50.0	0.001
600–1000	93	42.3	51	50.0	42	35.6	
≥ 1000	42	19.1	25	24.5	17	14.4	
**Time elapsed since diagnosis (years)**							
< 7	104	47.3	48	47.1	56	47.5	1.000
≥ 7	116	52.7	54	52.9	62	52.5	
**Median (Q1, Q3)**			7.0 (4.0, 15.0)		7.0 (4.0, 13.0)		0.442
**Has a health insurance**							
Yes	175	79.5	81	79.4	94	79.7	1.000
No	45	20.5	21	20.6	24	20.3	
**Has attended educational program regarding diabetes**							
Yes	22	10.0	6	5.9	16	13.6	0.072
No	198	90.0	96	94.1	102	86.4	
**Fasting plasma glucose (mg/dL)**							
< 140	66	30.0	29	28.4	37	31.4	0.661
≥ 140	154	70.0	73	71.6	81	68.6	
**Median (Q1, Q3)**			150.0 (120.0, 180.0)		150.0 (130.0, 180.0)		0.703
**Postprandial plasma glucose (mg/dL)**							
< 200	43	19.5	17	16.7	26	22.0	0.394
≥ 200	177	80.5	85	83.3	92	78.0	
**Median (Q1, Q3)**			240.0 (200.0, 300.0)		220.0 (200.0, 250.0)		0.185
**HbA**_**1c**_ **(%)**							
< 7	53	24.1	23	22.5	30	25.4	0.639
≥ 7	167	75.9	79	77.5	88	74.6	
**Median (Q1, Q3)**			7.8 (7.0, 8.8)		7.5 (7.0, 8.5)		0.467
**Body Mass Index (kg/m**^**2**^**)**							
< 25	45	20.5	20	19.6	25	21.2	0.867
≥ 25	175	79.5	82	80.4	93	78.8	
**Median (Q1, Q3)**			27.9 (25.7, 31.8)		29.3 (25.4, 33.3)		0.464

*HbA*_*1c*_ Glycated hemoglobin, Q1: 1st quartile, Q3: 3rd quartile, Median = Q2

### Knowledge, attitude, and practice of the patients with regard to their T2DM

Knowledge, attitude, and practice of the patients with regard to their T2DM were measured using relevant items. Detailed responses of the patients overall and stratified by their gender are shown in Table 2.

**Table 2:** Responses of the patients on the knowledge, attitude, and practice items overall and stratified by gender (*n = 220*)

#	Item	Total		Male		Female		***p***-value
		N	%	n	%	n	%	
**Knowledge**								
**1**	**Tests used to diagnose T2DM**							
	Blood tests	220	100.0	102	100.0	118	100.0	1.000
	Urine tests	48	21.8	15	14.7	33	28.0	0.022
**2**	**Keeping blood glycemia under control**							
	Taking medications (insulin and/or other oral hypoglycemic agents)	200	90.9	94	92.2	106	89.8	0.641
	Adhering to diet control	174	79.1	83	81.4	91	77.1	0.507
	Performing regular exercise	101	45.9	50	49.0	51	43.2	0.418
	Weight management	48	21.8	21	20.6	27	22.9	0.745
	Going for regular checkups	25	11.4	12	11.8	13	11.0	1.000
	I don’t know	7	3.2	3	2.9	4	3.4	1.000
**3**	**For how long should the patient adhere to diet control and/or taking medications**							
	Until blood sugar levels become under control	85	38.6	36	35.3	49	41.5	0.405
	Lifelong	135	61.4	66	64.7	69	58.5	0.405
**4**	**Major organs of the body are affected by T2DM**							
	Kidneys	124	56.4	61	59.8	63	53.4	0.344
	Feet	98	44.5	43	42.2	55	46.6	0.587
	Eyes	129	58.6	65	63.7	64	54.2	0.171
	Nerves	102	46.4	49	48.0	53	44.9	0.685
	Heart	82	37.3	40	39.2	42	35.6	0.675
	I don’t know	21	9.5	9	8.8	12	10.2	0.820
	**Knowledge score, Median (Q1, Q3)**			6.5 (4.5, 7.5)		5.5 (4.5, 7.5)		0.186
**Attitude**								
**1**	**It is alright to eat sweets on occasional basis**							
	Agree/undecided	80	36.4	34	33.3	46	39.0	0.402
	Disagree	140	63.6	68	66.7	72	61.0	
**2**	**It is alright if you forget to take your insulin and/or other antidiabetic agents on some days**							
	Agree/undecided	64	29.1	28	27.5	36	30.5	0.657
	Disagree	156	70.9	74	72.5	82	69.5	
**3**	**Patients with T2DM should visit their healthcare providers for regular checkups even if their blood glycemia is under control**							
	Disagree/undecided	64	29.1	30	29.4	34	28.8	1.000
	Agree	156	70.9	72	70.6	84	71.2	
**4**	**It is alright not to perform regular exercise as prescribed by the healthcare provider because patients exercise while performing daily activities**							
	Agree/undecided	97	44.1	39	38.2	58	49.2	0.134
	Disagree	123	55.9	63	61.8	60	50.8	
	**Attitude score, Median (Q1, Q3)**			3.0 (2.0, 4.0)		3.0 (2.0, 4.0)		0.288
**Practice**								
**1**	**Are you taking your insulin and/or antidiabetic agents as advised by your caring physician**							
	Yes	157	71.4	71	69.6	86	72.9	0.654
	No	63	28.6	31	30.4	32	27.1	
**2**	**Are you following the diet schedule recommended to you by your healthcare providers**							
	Yes	107	48.6	50	49.0	57	48.3	1.000
	No	113	51.4	52	51.0	61	51.7	
**3**	**Are you performing the regular exercises recommended to you by your healthcare providers**							
	Yes	82	37.3	41	40.2	41	34.7	0.485
	No	138	62.7	61	59.8	77	65.3	
**4**	**Is your blood glycemia under control**							
	Yes	105	47.7	46	45.1	59	50.0	0.637
	No	75	34.1	35	34.3	40	33.9	
	I am not sure	40	18.2	21	20.6	19	16.1	
**5**	**Do you visit your healthcare providers for regular checkups**							
	Yes	144	65.5	63	61.8	81	68.6	0.321
	No	76	34.5	39	38.2	37	31.4	
	**Practice score, Median (Q1, Q3)**			3.0 (1.0, 4.0)		3.0 (1.5, 4.0)		0.816

Q1: 1st quartile, Q3: 3rd quartile, Median = Q2

The median knowledge score of the patients was 6.0 (4.5, 7.5). When asked to identify the organs affected by DM, 129 (58.6%) could identify that DM could affect the eyes and 124 (56.4%) could identify that DM could affect the kidneys. Of the patients, only 82 (37.3%) could identify that DM could affect the heart (Table 3). When asked about how diabetes could be kept under control, the vast majority (90.9%) of the patients could identify medications including insulin. Diet was identified by 174 (79.1%) patients. However, going for regular checkups was identified by only 25 (11.4%) patients (Table 2). Distribution of responses were comparable between male and female patients. However, more female patients identified urine tests (*p*-value = 0.022) compared to male patients.

**Table 3:** Correlation between knowledge, attitude, and practice scores with socioeconomic and clinical variables of the patients

Variable		Knowledge score	Attitude score	Practice score	Age	Monthly household income	Time elapsed since diagnosis	Fasting plasma glucose	Postprandial plasma glucose	HbA_**1c**_	Body Mass Index
**Knowledge score**	**rho**	–	0.34	0.46	−0.26	0.27	−0.19	−0.24	−0.18	−0.17	−0.13
	***p*-value**		< 0.001	< 0.001	< 0.001	< 0.001	0.004	< 0.001	0.007	0.015	0.056
**Attitude score**	**rho**	0.34	–	0.49	0.00	0.08	0.00	−0.24	−0.24	−0.14	−0.19
	***p*-value**	< 0.001		< 0.001	0.958	0.221	0.997	< 0.001	< 0.001	0.048	0.005
**Practice score**	**rho**	0.46	0.49	–	− 0.24	0.11	− 0.30	− 0.30	− 0.36	− 0.38	− 0.24
	***p*-value**	< 0.001	< 0.001		< 0.001	0.116	< 0.001	< 0.001	< 0.001	< 0.001	< 0.001

*HbA*_*1c*_ Glycated hemoglobin

The median score of the patient attitude was 3.0 (2.0, 4.0). Of the patients, about 36% of patients believed that eating sweets occasionally was alright, about 29% of the patients stated that it was alright if they forgot to take their medicines on some days, around 30% of the patients believed that it was not necessary to go for regular checkups if their sugar level was under control, and close to 44% believed that they got enough exercise when doing their daily activity (Table 2). Distribution of responses were comparable between male and female patients (*p*-value > 0.05).

The median practice score of the patients was 3.0 (1.0, 4.0). Of the patients, about 71% stated that they regularly took their insulin and/or antidiabetic medications as advised by their physicians, about 39% followed the diet program as advised by their healthcare providers, approximately 37% stated that they adhered to regular exercises as advised by their healthcare providers, and around 65% stated that they regularly go for follow ups (Table 2). Distribution of responses were comparable between male and female patients (*p*-value > 0.05).

There were no statistically significant differences (*p*-value > 0.05) between the overall knowledge, attitude, and practice scores of male and female patients. Details of the knowledge, attitude, and practice scores of the patients stratified by their gender are shown in Table 2.

### Association and correlation between sociodemographic and clinical characteristics of the patients with knowledge, attitude, and practice scores

When the continuous variables were correlated, there was moderate positive correlation between knowledge, attitude, and practice scores (*p*-value < 0.001) scores. Details of these correlations are shown in Table 3.

Knowledge scores correlated positively with monthly income (Spearman’s rho = 0.27, *p*-value < 0.001) and negatively with age (Spearman’s rho = − 0.26, *p*-value < 0.001), time elapsed since diagnosis (Spearman’s rho = − 0.19, *p*-value < 0.001), fasting plasma glucose (Spearman’s rho = − 0.24, *p*-value < 0.001), postprandial glucose (Spearman’s rho = − 0.18, *p*-value < 0.001), and HbA_1c_ (Spearman’s rho = − 0.17, *p*-value = 0.015). Details of the correlations are shown in Table 3. The median knowledge score was significantly higher for patients who were younger than 55 years old (*p*-value < 0.001), single (*p*-value = 0.021), employed (*p*-value = 0.001), had university education (*p*-value < 0.001), had higher monthly income (*p*-value < 0.001), diagnosed with T2DM since less than 7 years (*p*-value = 0.001), had usual fasting plasma glucose of less than 140 mg/mL (*p*-value = 0.003), had HbA_1c_ of less than 7% (*p*-value = 0.037), and had a BMI of less than 25 kg/m^2^ (*p*-value = 0.039). Details of the differences are shown in Table 4.

**Table 4:** Differences in knowledge, attitude, and practice scores among the patients stratified by their sociodemographic and clinical variables

Variable	n	%	Knowledge		Attitude		Practice	
			Median (Q1, Q3)	***p***-value	Median (Q1, Q3)	***p***-value	Median (Q1, Q3)	***p***-value
**Age (years)**								
< 55	82	37.3	6.5 (5.5, 8.5)	< 0.001	3.0 (2.0, 4.0)	0.979	4.0 (2.0, 4.0)	< 0.001
≥ 55	138	62.7	5.5 (3.5, 7.5)		3.0 (2.0, 4.0)		3.0 (1.0, 4.0)	
**Gender**								
Male	102	46.4	6.5 (4.5, 7.5)	0.186	3.0 (2.0, 4.0)	0.288	3.0 (1.0, 4.0)	0.816
Female	118	53.6	5.5 (4.0, 7.5)		3.0 (2.0, 4.0)		3.0 (1.8, 4.0)	
**Marital status**								
Single	32	14.5	7.3 (6.1, 8.5)	0.021	2.5 (2.0, 4.0)	0.722	3.0 (1.0, 5.0)	0.561
Married	156	70.9	5.5 (4.5, 7.5)		3.0 (2.0, 4.0)		3.0 (2.0, 4.0)	
Divorced/widowed	32	14.5	6.5 (3.5, 8.8)		3.0 (2.0, 4.0)		3.0 (1.0, 3.8)	
**Employment**								
Unemployed	139	63.2	5.5 (4.0, 7.50	0.001	3.0 (2.0, 4.0)	0.659	3.0 (1.0, 4.0)	0.230
Employed	81	36.8	6.5 95.5, 8.3)		3.0 (2.0, 4.0)		3.0 (1.0, 4.0)	
**Place of residence**								
Urban	149	67.7	6.5 (4.5, 7.5)	0.520	3.0 (2.0, 4.0)	0.853	3.0 (1.0, 4.0)	0.863
Country side	71	32.3	5.5 (4.5, 7.5)		3.0 (2.0, 4.0)		3.0 (2.0, 4.0)	
**Educational level**								
Some school	165	75	5.5 (4.0, 7.0)	< 0.001	3.0 (2.0, 4.0)	0.253	3.0 (1.0, 4.0)	0.001
University	55	25	7.5 (5.5, 10.0)		3.0 (2.0, 4.0)		4.0 (2.0, 5.0)	
**Monthly household income (Jordanian Dinar)**								
< 600	85	38.6	5.5 (4.0, 6.5)	< 0.001	2.0 (2.0, 4.0)	0.290	3.0 (1.0, 4.0)	0.192
600–1000	93	42.3	6.5 (5.0, 7.5)		3.0 (2.0, 4.0)		3.0 (1.0, 4.0)	
≥ 1000	42	19.1	7.5 (4.5, 9.5)		3.0 (1.8, 4.0)		3.0 (2.0, 4.0)	
**Time elapsed since diagnosis (years)**								
< 7	104	47.3	6.5 (5.0, 8.5)	0.001	3.0 (2.0, 4.0)	0.892	4.0 (2.0, 5.0)	< 0.001
≥ 7	116	52.7	5.5 (4.0, 7.5)		3.0 (2.0, 4.0)		3.0 (0.3, 3.0)	
**Has a health insurance**								
Yes	175	79.5	6.0 (4.5, 7.5)	0.230	3.0 (2.0, 4.0)	0.255	3.0 (1.0, 4.0)	0.257
No	45	20.5	6.5 (4.5, 8.3)		3.0 (2.0, 4.0)		3.0 (1.0, 5.0)	
**Has attended educational program regarding diabetes**								
Yes	22	10	6.0 (4.5, 7.5)	0.969	3.0 (2.0, 4.0)	0.547	3.5 (3.0, 4.0)	0.026
No	198	90	6.5 (4.5, 7.5)		3.0 (2.0, 4.0)		3.0 (1.0, 4.0)	
**Fasting plasma glucose (mg/dL)**								
< 140	66	30	6.5 (5.0, 8.5)	0.003	3.0 (2.0, 4.0)	0.013	4.0 (2.0, 5.0)	< 0.001
≥ 140	154	70	5.5 (4.5, 7.5)		3.0 (2.0, 4.0)		3.0 (1.0, 4.0)	
**Postprandial plasma glucose (mg/dL)**								
< 200	43	19.5	6.5 (4.0, 8.5)	0.423	3.0 (2.0, 4.0)	0.114	4.0 (3.0, 5.0)	< 0.001
≥ 200	177	80.5	6.0 (4.5, 7.5)		3.0 (2.0, 4.0)		3.0 (1.0, 4.0)	
**HbA**_**1c**_ **(%)**								
< 7	53	24.1	6.5 (4.8, 9.5)	0.037	3.0 (2.0, 4.0)	0.301	4.0 (3.0, 4.0)	0.001
≥ 7	167	75.9	5.5 (4.5, 7.5)		3.0 (2.0, 4.0)		3.0 (1.0, 4.0)	
**Body Mass Index (kg/m**^**2**^**)**								
< 25	45	20.5	6.5 (5.0, 8.5)	0.039	3.0 (2.0, 4.0)	0.211	4.0 92.5, 5.0)	0.001
≥ 25	175	79.5	5.5 (4.5, 7.5)		3.0 (2.0, 4.0)		3.0 (1.0, 4.0)	

*HbA*_*1c*_ Glycated hemoglobin, Q1: 1st quartile, Q3: 3rd quartile, Median = Q2

Attitude scores correlated negatively with fasting plasma glucose (Spearman’s rho = −0.24, *p*-value < 0.001), postprandial plasma glucose (Spearman’s rho = −0.24, *p*-value < 0.001), HbA_1c_ (Spearman’s rho = −0.14, *p*-value = 0.048), and BMI (Spearman’s rho = −0.19, *p*-value = 0.005). Details of the correlations are shown in Table 3. The median attitude score was significantly (*p*-value = 0.013) higher for patients who had usual fasting glucose levels of less than 140 g/dL compared to patients who had usual fasting glucose levels of 140 g/dL and more. Details of the differences are shown in Table 4.

Practice scores correlated negatively with age (Spearman’s rho = − 0.24, *p*-value < 0.001), time elapsed since diagnosis (Spearman’s rho = − 0.30, *p*-value < 0.001), fasting plasma glucose (Spearman’s rho = − 0.30, *p*-value < 0.001), postprandial plasma glucose (Spearman’s rho = − 0.36, *p*-value < 0.001), HbA_1c_ (Spearman’s rho = − 0.38, *p*-value < 0.001), and BMI (Spearman’s rho = − 0.24, *p*-value < 0.001). Details of the correlations are shown in Table 3. The median practice score was significantly higher for patients who were younger than 55 years (*p*-value < 0.001), had university education (*p*-value = 0.001), were diagnosed since less than 7 years (*p*-value < 0.001), had attended an educational program on diabetes (*p*-value < 0.001), had usual fasting glucose levels of less than 140 g/dL (*p*-value < 0.001), had usual postprandial plasma glucose of less than 200 g/dL (*p*-value < 0.001), had HbA_1c_ < 7% (*p*-value = 0.001), and had a BMI < 25 kg/m^2^ (*p*-value = 0.001). Details of the differences are shown in Table 4.

### Predictors of higher knowledge, attitude, and practice scores

In this study, a multiple linear regression model was used to control confounding variables and to identify predictors of higher knowledge, attitude, and practice scores. The R^2^ of the model was 0.22 with a *p*-value of < 0.001. The model showed that having university education was strongly associated with having higher knowledge scores (*p*-value = 0.001). Additionally, the model showed that having attended an educational program on diabetes was moderately associated with higher practice scores (*p*-value = 0.026). Details of the multiple linear regression model are shown in Table 5.

**Table 5:** Multiple linear regression between sociodemographic and clinical variables of the patients with knowledge, attitude, and practice scores

Variable	Unadjusted Coefficients	SE	Adjusted Coefficients	t	***p***-value
**Knowledge score**					
Gender	−0.03	0.38	−0.01	− 0.07	0.942
Marital status	−0.01	0.32	0.00	−0.03	0.979
Employment	0.13	0.44	0.03	0.30	0.762
Place of residence	−0.18	0.35	−0.03	− 0.51	0.609
Educational level	1.47	0.42	0.27	3.49	0.001
Monthly income	0.43	0.25	0.13	1.71	0.088
Having a health insurance	−0.40	0.40	− 0.07	−1.02	0.307
Having attended an educational program	0.23	0.55	0.03	0.42	0.676
Age	−0.04	0.02	−0.17	−1.82	0.070
Time elapsed since diagnosis	0.03	0.03	0.09	0.97	0.332
Body Mass Index	0.00	0.03	0.00	0.01	0.990
Fasting plasma glucose	−0.01	0.01	−0.19	−1.55	0.123
Postprandial plasma glucose	0.00	0.01	−0.02	− 0.14	0.887
HbA_1c_	0.10	0.18	0.06	0.57	0.568
**Attitude score**					
Gender	−0.20	0.21	− 0.08	− 0.96	0.338
Marital status	0.11	0.18	0.05	0.60	0.552
Employment	0.01	0.25	0.00	0.04	0.966
Place of residence	−0.08	0.20	−0.03	−0.40	0.687
Educational level	0.12	0.24	0.04	0.50	0.617
Monthly income	0.00	0.14	0.00	−0.02	0.982
Having a health insurance	−0.13	0.22	−0.04	− 0.59	0.554
Having attended an educational program	0.12	0.31	0.03	0.40	0.689
Age	0.00	0.01	0.00	−0.03	0.977
Time elapsed since diagnosis	0.01	0.02	0.08	0.80	0.426
Body Mass Index	−0.02	0.02	−0.09	−1.25	0.211
Fasting plasma glucose	0.00	0.00	−0.01	−0.04	0.964
Postprandial plasma glucose	−0.01	0.00	−0.30	−1.86	0.064
HbA_1c_	0.08	0.10	0.09	0.79	0.432
**Practice score**					
Gender	−0.16	0.26	− 0.05	− 0.62	0.536
Marital status	−0.05	0.22	−0.02	− 0.24	0.813
Employment	−0.26	0.30	− 0.08	− 0.86	0.393
Place of residence	0.03	0.24	0.01	0.14	0.891
Educational level	0.35	0.29	0.09	1.20	0.232
Monthly income	−0.03	0.17	−0.01	− 0.15	0.882
Having a health insurance	−0.14	0.27	−0.03	− 0.53	0.599
Having attended an educational program	0.85	0.38	0.15	2.24	0.026
Age	−0.01	0.01	−0.09	− 0.93	0.353
Time elapsed since diagnosis	−0.02	0.02	−0.09	− 0.97	0.333
Body Mass Index	−0.03	0.02	−0.11	−1.57	0.119
Fasting plasma glucose	0.01	0.01	0.13	1.07	0.287
Postprandial plasma glucose	−0.01	0.00	−0.28	−1.84	0.068
HbA_1c_	− 0.15	0.12	− 0.13	−1.19	0.234

*HbA*_*1c*_ Glycated hemoglobin, *SE* standard error, *t* t statistic

## Discussion

In this cross-sectional study, knowledge, attitude, and practice of patients with T2DM with regard to their disease were assessed for the first time among Palestinian patients. Patients who took part in this study were recruited from different primary healthcare facilities in the West Bank of Palestine. In this study, more than half (52.2%) had good knowledge and 58.7% has positive attitude with regard to their disease. On the other hand, only 36.4% had good practice. Findings of this study highlighted gaps in knowledge, attitude, and practice with regard to T2DM among patients. In this study, associations and correlations were established between knowledge, attitude, and practice with the sociodemographic and clinical characteristics of the patients. Additionally, predictors of higher knowledge, positive attitude, and good practice were identified. Findings of this study could be useful for policy makers, decision makers, healthcare providers, and patient advocacy groups who might need to design interventions to improve health outcomes of patients with T2DM.

Interestingly, there was a moderate positive correlation between knowledge, attitude, and practice scores. This could at least in part support the argument that good knowledge might promote positive attitude and adequate practice among patients with T2DM [19–21]. Our findings indicated that almost half of the patients (52.2%) had good knowledge of T2DM and its associated complications. Patients who were young than 55 years, single, had a university education, employed, had higher income, diagnosed since less than 7 years, had usual fasting plasma glucose of less than 140 mg/mL, had HbA_1c_ of less than 7%, and had a BMI of less than 25 kg/m^2^ scored higher in the knowledge test. In a pilot study conducted in Sri Lanka, Hearth et al. showed that 77% of patients with T2DM has moderate or above moderate knowledge of their disease [3]. Studies in different settings have reported variable level of knowledge among patients with T2DM in Mongolia, Sri Lanka, Bangladesh, India, Jordan, and Lebanon [35–41]. When we controlled potential confounding variables, having university education was strongly associated with having higher knowledge scores. Findings of this study might indicate that educated patients are more likely to be more aware of their disease, its complications, and ways to keep their blood glucose under control. Additionally, it has been argued that university educated patients are more likely to be interested in learning about their disease compared to uneducated patients who might be less interest in learning about their diseases. Taken together, our findings might suggest that less educated patients need greater motivation and support from their healthcare providers and families. Our findings were consistent with those reported in previous studies that were conducted elsewhere [3, 36, 37]. For example, Hearth et al. showed that education was positively associated with higher knowledge of T2DM among patients in Sri Lanka [3]. Similarly, Karaoui et al. showed that higher level of education was positively correlated with higher knowledge of T2DM among patients in Lebanon [37]. In Bangladesh, Fatema et al. showed that male patients with T2DM had significantly higher knowledge of their disease compared to female patients [36].

Findings of this study showed that 58.7% of the patients had positive attitudes toward their disease. Attitude scores were positively associated with ability to keep a usual fasting plasma glucose level below 140 mg/dL, postprandial plasma glucose level below 200 mg/dL, HbA_1c_ below 7%, and BMI below 25 kg/m^2^. When we controlled potential confounding variables, attitude scores were no longer associated with these variables. Findings of this study were contradictory to those reported by Belsti et al. in Ethiopia in which attitudes of the patients were associated with their educational level and income [26]. Previous studies suggested that patients with higher income could have better access to healthcare services, ability to go to regular checkups, and practice physical activity compared to patients with less income [25, 36].

Regarding practice, our study showed that 36.4% of the patients with T2DM had good practices with regard to their disease. Patients who were younger than 55 years, had university education, were diagnosed since less than 7 years, had attended an educational program on diabetes, had a usual fasting glucose levels of less than 140 g/dL, had postprandial plasma glucose of less than 200 g/dL (*p*-value < 0.001), had HbA_1c_ of less than 7%, and had a BMI of less than 25 kg/m^2^ reported higher practice scores. Our results were consistent with those reported in different settings in Ethiopia, Mongolia, Sri Lanka, Bangladesh, and Lebanon [3, 26, 35–37]. When we controlled potential confounding variables, having attended an educational program on diabetes was moderately associated with higher practice scores. Our findings might support previous findings on the effectiveness of educational programs in improving self-management and health outcomes in patients with T2DM [42, 43].

### Strengths and limitations

Results of this study might be carefully interpreted taking into consideration the following strengths and limitations. First, this study was the first to assess knowledge, attitude, and practice of Palestinians with T2DM with regard to their disease. The tool used to measure knowledge, attitude, and practice was adopted from previous studies [24–26]. The tool was piloted for clarity and comprehension. Additional face validity, testing for stability of scores over a short period of time, and internal consistency between the items included were performed. The test-retest method and Cronbach’s alpha ensured that the tool used in this study was reliable and internally consistent [28–32]. These measures might have added strength and rigor to methods used in this study. Second, the data were collected using an interviewer administered questionnaire. Additionally, the interviewers in this study were final year MD candidates who were familiar with conducting interviews and taking patient history. This could have minimized occasions of mis- or lack of understanding associated with self-administered questionnaires [44]. Third, this study also established associations and correlations between knowledge, attitude, and practice with various sociodemographic and clinical characteristics of the patients. Fourth, predictors of higher knowledge, attitude, and practice scores were also identified in this study. Fifth, the study tool used in this study was piloted and evaluated for reliability and internal consistency. Finally, the study tool was administered by interviewers who were final year MD candidates who were familiar with interviewing patients and taking medical history. This could have reduced the occasions of mis- or lack of understanding that could be associated with self-administered questionnaires.

On the other hand, this study has a number of limitations. First, this study was a cross-sectional study. An interventional design could have permitted enhancing knowledge, improved positive attitude, and promoted good practice among patients with T2DM with regard to their disease. Second, the sample size used in this study was relatively small. However, the sample size used in this study was comparable to those used in other studies [3, 37]. Third, a convenience sampling method was followed to recruit the sample needed for this study. It is noteworthy mentioning that the sample recruited in this study was diversified by inclusion of patients from both genders, different educational levels, income levels, and geographic locations. Finally, the number of items relevant to knowledge, attitude, and practice was relatively small. Despite the small number of items, we were able to expose the level of knowledge, attitude, and practice of patients with T2DM with regard to their disease.

## Conclusion

In conclusion, this study provided insights into knowledge, attitude, and practice of Palestinians with T2DM. The study established association and correlation between knowledge, attitude, and practice scores with sociodemographic and clinical characteristics of patients with T2DM in Palestine. Findings of this study highlighted the need for appropriately designed interventions to increase knowledge about T2DM among patients with low educational level. Well-designed educational programs might promote healthy practice among patients with T2DM. Future studies are still needed to assess if such interventions could be effective in improving health outcomes and quality of life of patients with T2DM in Palestine.

## Data Availability

The datasets used and/or analyzed during the current study are available from the corresponding author on reasonable request.

## Abbreviations

BMI: Body mass index
T2DM: Type 2 diabetes mellitus
HbA_1c_: Glycated hemoglobin
IRB: Institutional Board Review
MD: Doctor of Medicine
Q1: 1st quartile
Q2: 2nd quartile (median)
Q3: 3rd quartile
